# Long-Time Scale Simulations Reveal Key Dynamics That Drive the Onset of the N State in the Proteorhodopsin Photocycle

**DOI:** 10.1021/acs.jpcb.4c02855

**Published:** 2024-10-10

**Authors:** Kyle R. Billings, Sadegh Faramarzi, Blake Mertz

**Affiliations:** † Food and Drug Administration, Frederick, Maryland 21701, United States; ‡ C. Eugene Bennett Department of Chemistry, West Virginia University, Morgantown, West Virginia 26506, United States; § Alivexis, Cambridge, Massachusetts 02142, United States

## Abstract

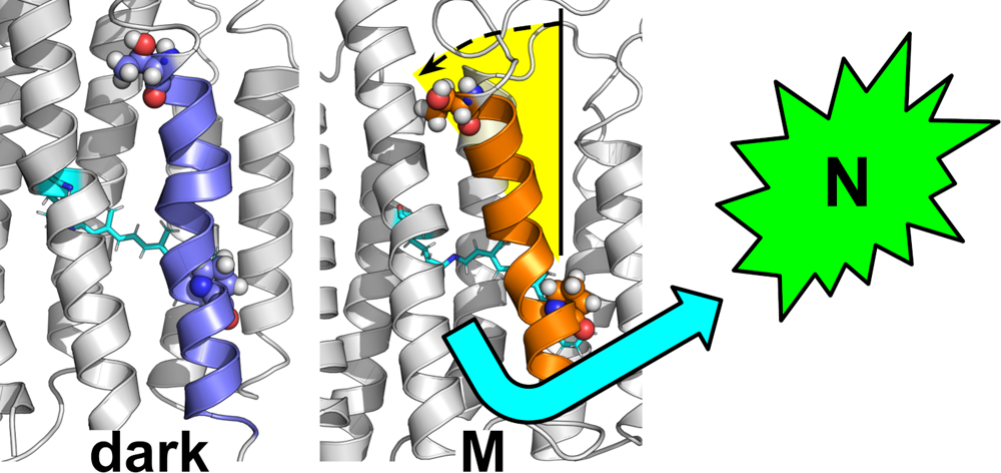

Proteorhodopsin (PR) is a microbial proton pump that plays a significant role in phototrophy of bacteria in marine environments. Fundamental understanding of the structure–function relationship that drives proton pumping in PR has largely been elusive due to a lack of high-resolution structures of the photointermediates in the PR photocycle. Extending upon previous work, we used long-time scale molecular dynamics (MD) simulations to characterize the M state of the blue variant of PR, which represents the first proton transfer that takes place in the photocycle. Several notable structural changes occur in the M state that are hallmarks of subsequent steps in the PR photocycle, indicating that although this protein is often compared to the canonical microbial rhodopsins, such as bacteriorhodopsin, PR possesses characteristics that make it distinct among the rapidly increasing and widely variable catalog of microbial rhodopsins.

## Introduction

Sunlight is our most abundant biologically available energy source, making solar energy harvesting evolutionally advantageous for microbes. One of the most common mechanisms for light harvesting is via microbial rhodopsins (MRs), and the number and scope of function of MRs has vastly expanded over the past two decades.^1,2^ Proteorhodopsin (PR), the MR that spawned the field of metagenomics,^1,3^ is primarily found in marine environments but has been observed in terrestrial bacteria, freshwater bacteria, viruses, and even eukaryotes,^4^ indicating that horizontal gene transfer of PRs provides an evolutionary advantage. The full extent of the biological function PR is unknown, but evidence suggests that PR is a critical workhorse for microbes, providing alternative sources of chemical energy in periods of nutrient deficiency, playing a role in degradation of organic matter in the oceanic carbon cycle, and driving the majority of phototrophy in marine environments.^5−8^

Like all microbial rhodopsins, PR consists of seven transmembrane (TM) helices with a retinal chromophore covalently bound to a lysine residue on helix F via a Schiff base (SB) linkage.^9^ Activation of PR occurs via all-*trans* → 13-*cis* isomerization that is driven by absorption of a photon. This photoactivation drives PR through a photocycle with five spectroscopically distinct photointermediates (i.e., states) that are also characterized by proton transfers that lead to pumping of a proton across the cell membrane from the cytoplasm to the periplasm.^10^ Specifically, the dark state is characterized by a protonated SB. Photoisomerization rearranges the conformation of the retinal pocket (dark K state), leading to a proton transfer from the SB to the proton acceptor, D97 (K M state). Transition from the M N state is characterized by reprotonation of the SB via the proton donor, E108, and subsequent uptake of a proton from bulk cytoplasm (N O). Completion of the PR photocycle occurs when a proton is released into bulk periplasm by the putative proton release group, E142, D97 donates its proton to the proton release group of PR, and retinal reverts back to the all-*trans* conformation (Figure S4).^10^ Two light-adapted variants of PR exist (green and blue PR) that are mainly distinguished by a color-tuning residue at position 105.^11,12^

Although other MRs like channelrhodopsin have been adopted as the protein of choice for applications in optogenetics,,^13^ PR is a compelling protein for obtaining fundamental understanding of MRs due to its biological role and the ability to easily manipulate the PR photocycle and oligomerization.^14−17^ Spectroscopic studies have revealed many aspects of the PR photocycle,^18−20^ but crystallography has been limited to the ground state structures of PR.^9,21^ Molecular dynamics (MD) simulations have been an invaluable tool in uncovering the structure–function relationship of rhodopsins, providing an atomistic level of detail to describe the dynamics of the retinal chromophore,^22^ the mechanism behind large-scale conformational changes in the protein,^23^ and the role of the membrane environment on protein function.^17^ Building on our previous studies characterizing the ground and K states of blue PR,^24,25^ we carried out long time scale simulations modeling the M state of PR. Surprisingly, we observed several structural and dynamical changes within the protein that are hallmarks of the N state, indicating that the transition of PR from the M to the N state is a series of steps rather than simply reprotonation of the Schiff base.

## Methods

### System Setup

Starting coordinates for blue PR were obtained (PDB 4JQ6),^9^ and missing residues were either modeled in with CHARMM or for loops >20 residues with corresponding coordinates from the NMR structure of green PR (PDB 2L6X).^26^ Protonation states for all titratable residues were assigned assuming pH of 7.0 unless otherwise specified (see Table S4). PR was placed inserted into a bilayer of 160 lipid molecules (80 per leaflet) with a molar ratio of 3:1 1-palmitoyl-2-oleoyl-*sn*-glycero-3-phosphoethanolamine/1-palmitoyl-2-oleoyl-*sn*-glycero-3-phosphoglycerol as a mimic of Gram-negative bacteria using the replacement method of the CHARMM-GUI server.^27^ Final system sizes after solvation and ionization were approximately 41,000 atoms. Equilibration of all dark state systems were carried out in NAMD 2.10^28^ in the *NPT* ensemble (*T* = 310 K, *P* = 1 atm) for 100 ns using a 2 fs time step. Details can be found in Feng and Mertz.^25^ The c36 CHARMM force field parameters were used for proteins, ions, and lipids,^29,30^ TIP3P force field for water molecules,^31^ and CHARMM-compatible force field for both protonation states of the retinal.^32,33^ The dark state has a 3 μs of production of each trajectory, and snapshots were used as the starting point for K intermediate simulations. Induction of the all-*trans* → 13-*cis* isomerization was induced by a temporary reduction of the torsional energy barrier about the C13=C14 bond, after which each K state simulation ran for 12 μs.^25^ A total of 4 representative snapshots from the K state simulations were then used as starting coordinates for the M state simulations. To represent the M state, the proton from the Schiff base was moved to the side chain of the proton acceptor D97 using topoTools and psfgen.^34,35^ All equilibrium MD simulations were run with a 2 fs time step in the *NPT* ensemble at 310 K and 1 bar using the Langevin thermostat and the Berendsen barostat (relaxation time of 8 ps). A cutoff of 8 Å was applied for nonbonded interactions, and long-range electrostatics were calculated using the particle mesh ewald (PME) implementation in AMBER.

### Production MD Simulations

Equilibrium simulations of the M state structures were performed for 200 ns using the CHARMM36 force field in NAMD2.13 before converting coordinates to be compatible with AMBER. All equilibrium simulations were conducted in the *NPT* ensemble (*T* = 310 K and *P* = 1 atm) using the Nosé–Hoover thermostat and barostat. Production simulations used the pmemd.cuda GPU implementation in AMBER engine with the thermostat and barostat settings mentioned above to obtain independent 20 μs trajectories.^36^ Simulations were performed with a 2 fs time step, and snapshots were taken every 1 ns.

### Analysis

The trajectory format was converted from Amber netcdf to DCD format using traj2dcd in LOOS.^37,38^ All of the trajectories were recentered around the protein. Interatomic distances were taken using the minimal distance between the selected atom via interdist in LOOS.^37,38^ The number of waters within a protein was calculated by juxtaposition of a cylindrical slice along the backbone of the protein and counting the number of OH2 atoms within each slice using LOOS and PYLOOS.^37,38^ The kink of helix F was defined by finding the angle between residues 170–175 and residues 182–190 using the helix-kink tool in LOOS.^37,38^ The EF loop is defined from residues 150–170 and the secondary structure was calculated using the timeline plugin within vmd and the stride algorithm.^35^ A 50% helicity was used as the threshold to classify the EF loop in a coiled or helical conformation.

## Results and Discussion

**Figure 1:**
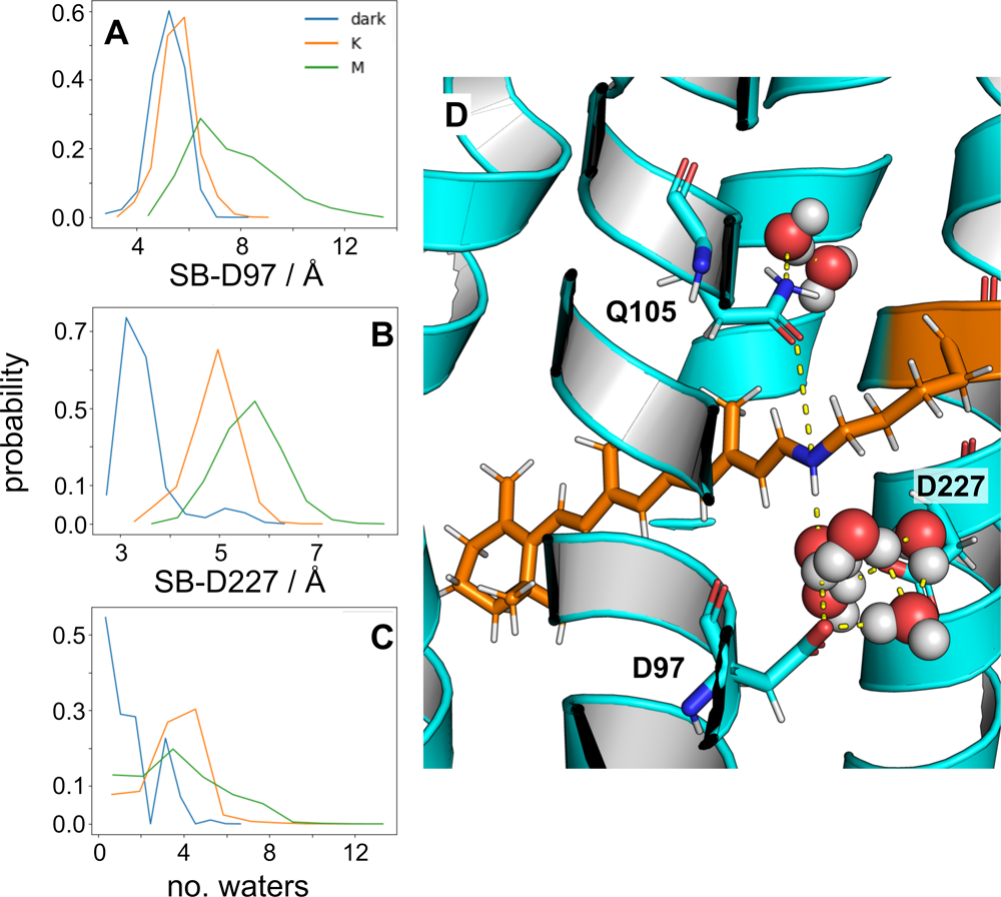
Protonation of the proton acceptor D97 disrupts the hydrogen-bonded network within the retinal binding pocket. (A) The probability distribution of distances between the N of the Schiff base to the side chain O of D97. (B) The probability distribution of distances between the SB N and the side chain O of D227. (C) The probability distribution of the number of waters within 5 Å of the carboxyl group of D97 and the NZ atom of RET. *Blue*: dark state; *orange*: K state; *green*: M state. (D) Representative snapshot of the retinal binding pocket in the dark state with residues that directly interact with the chromophore. *Orange sticks*: retinal and K231; *cyan sticks*: residues important to color-tuning (Q105) and proton transfer (D97 and D227); *spheres*: water molecules within the retinal binding pocket.

Destabilization of the interaction of D97 with the SB is dependent on the photointermediate (i.e., the protonation state of the binding pocket). The SB has direct interactions with both the proton acceptor, D97, and the complex counterion, D227, in the dark state (Figures 1A,B, S1, and S2).^20,39−41^ However, upon photoisomerization, a sequential shift in these direct interactions occurs, allowing the SB to utilize water-mediated interactions to facilitate proton transfer from the SB to D97. During progression to the M state, direct interactions between the SB and D97/D227 are abolished as more waters diffuse into the retinal binding pocket (Figures 1C, S4, and S5).^18^ Fourier transform infrared spectroscopy (FTIR) studies had previously shown this sequence of events within the retinal binding pocket of green PR.^42^ Our observations are consistent with the established mechanism of PR and microbial rhodopsins in general; water-mediated proton transfers are critical to progression through the photocycle. Depending on the function and structural arrangement of a given microbial rhodopsin, the number of waters that stabilize the SB complex varies between a diamond-shaped or pentagonal water cluster.^43,44^ A representative snapshot of the retinal binding pocket in the dark state shows that our MD simulations capture this geometry, with a cluster of five water molecules nested within the region between the SB, D97, and D227 (Figure 1D). To facilitate long-range proton transfer across the length of PR, it is necessary for a chain of water molecules to diffuse into the cytoplasmic and periplasmic half-channels. Based on our MD simulations, it appears that diffusion of water into the half-channels of PR occurs over longer (microsecond) time scales, although determining the rate-limiting step in the progression from the K to the M state (i.e., diffusion of water or structural rearrangements of PR) is a more complex issue that will be described below.

**Figure 2:**
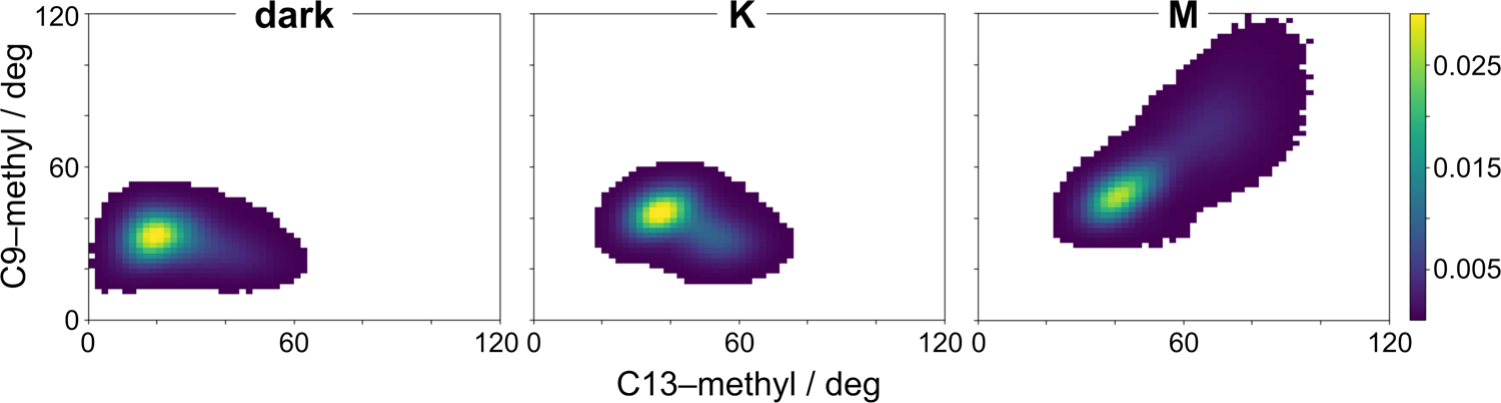
Shift in dynamics of the retinal polyene chain is a hallmark of the onset of the M state. Distribution of the angle of the C9-methyl with respect to the membrane normal versus the angle of the C13-methyl with respect to the membrane normal for the dark (*left*), K (*middle*), and M (*right*) states in the PR photocycle.

Knowing that the hydrogen-bonded network in the retinal binding pocket undergoes rearrangements in the transitions to the K and M states, we wanted to characterize the role of the retinal chromophore in this process. Previous studies have shown that the C9–and C13–methyl groups of the polyene chain are excellent reporters of the dynamics of retinal, both for the photoreceptor, rhodopsin,^45,46^ as well as for the canonical microbial proton pump, bacteriorhodopsin.^47−49^ In addition, the polyene methyl groups are critical to the function of microbial rhodopsins.^50−52^ We observe a very slight shift in the population of the C9–and C13–methyl orientations from the dark to the K state, but the overall distribution is constricted to a small conformational space indicating the stability of the polyene chain within the retinal binding pocket (Figure 2). However, upon proton transfer from the SB to D97 and onset of the M state, the polyene chain undergoes a more marked out-of-plane rotation (ϕ > 75°). This shift occurs in 1 of the 4 M state trajectories (Figures S6 and S7). Although this observation could be an outlier, the orientation of the polyene methyl groups remains stable for 20 μs, indicating that this trajectory may be capturing a viable transition. Based on our knowledge of the photocycle of other microbial rhodopsins (e.g., bacteriorhodopsin, channelrhodopsin), we know that transition from the K to the M and N states requires a rotation of the Schiff base away from the proton acceptor.^53,54^ In addition, previous NMR and flash photolysis studies on bR have shown that the retinal polyene chain can undergo rotation from 5 to 20° during the photocycle.^55−57^ The combination of these two phenomena opens up the possibility that the larger rotation of the polyene chain in one of our M state trajectories is a potential pathway to facilitate the reorientation of the SB toward the proton donor, E108, in the M → N transition.

**Figure 3:**
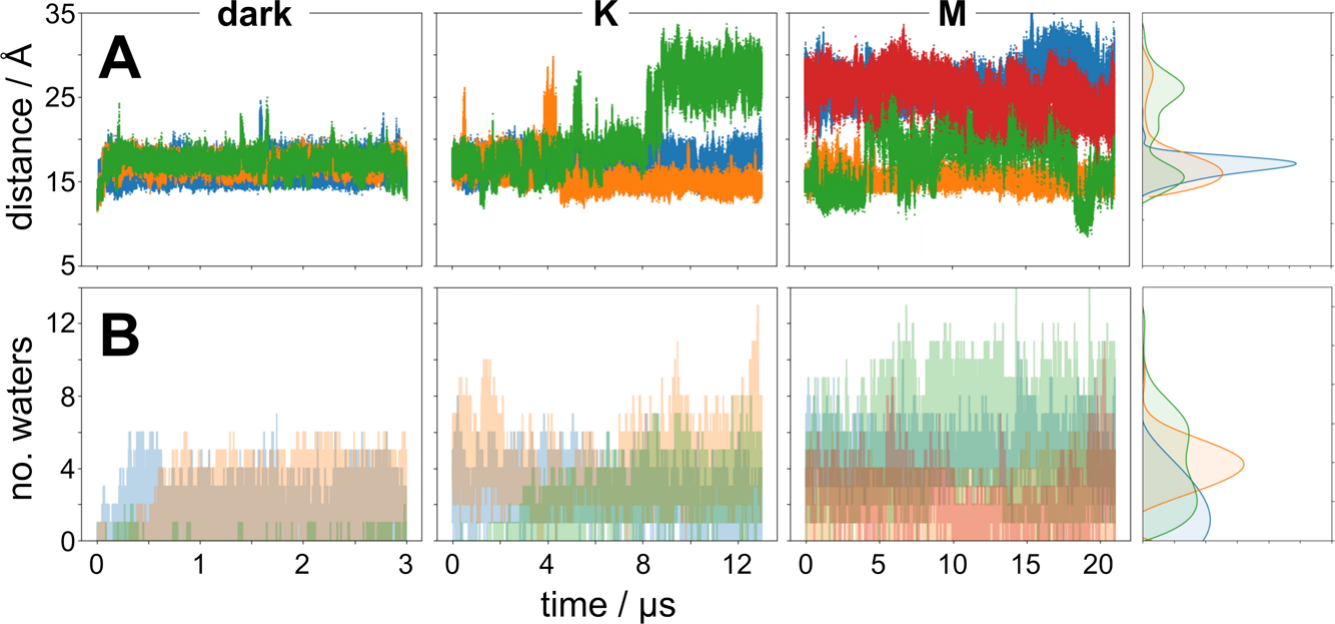
Position of E142 is correlated with internal hydration of the periplasmic half-channel. (A) Distance between side chain O of E142 and the SB N as a function of time for the dark (*left*), K (*middle*), and M (*right*) states. Different colors represent independent trajectories. (B) Number of water molecules in the periplasmic half-channel as a function of time for the dark (*left*), K (*middle*), and M (*right*) states. The number of water molecules is calculated by counting the number of water oxygens within an elliptical cylinder defined with a height of the distance between E142 atoms and the PSB. Different colors represent independent trajectories. *Far right*: projection of the probability density for each respective measurement for the dark (*blue*), K (*orange*), and M (*green*) states. Probability densities were rendered using a smoothing function in matplotlib.

The position of E142 with respect to the SB of retinal has good correlation with hydration of the periplasmic half channel of PR. For the majority of the time in the dark and K states, E142 is oriented toward the retinal binding pocket, effectively closing off the half-channel from bulk solvent in the periplasm. In one trajectory we observe an increase in distance in the K state, which occurs due to rotation of the E142 side chain away from the retinal binding pocket.^24^ However, upon proton transfer from the SB to D97 in the M state, in three of the four trajectories the long-range interaction between E142 and the SB either remains much weaker (around 25–30 Å) or transiently changes from its orientation in the dark and K states to one away from the binding pocket (Figure 3A). If the interaction is completely broken (distance >20 Å), this allows an influx of water into the periplasmic half-channel of PR to fully hydrate that portion of the protein (Figure 3B). Based on these observations, internal hydration of the periplasmic half of PR is presumably a two-step mechanism: photoisomerization of retinal rearranges the binding pocket, after which the change in the electrostatic environment of the binding pocket in the form of proton transfer from the SB to D97 weakens the interaction of E142 to the point that bulk solvent from the periplasm can freely diffuse into PR. This indicates a role for E142 as the putative proton release group in facilitating proton transfer from the retinal binding pocket to the periplasm.^9^ Structures of other microbial rhodopsins that are genetically similar to PR have a similar conformational arrangement in the region of the proton release group; both *E. sibiricum* rhodopsin (ESR) and xanthorhodopsin (XR) have a cavity in the periplasmic region of the protein that allows for direct contact with bulk solvent, where a single acidic residue (D214 in ESR and D90 in XR) serves as the access point for proton transfers to and from the retinal binding pocket.^58,59^

Another aspect of E142 that is unique to blue PR is the fact that it is deprotonated in the dark state, whereas in green PR E142 is protonated.^60^ The D97N mutation in green PR has been shown to block formation of the M photointermediate,^39^ whereas in blue PR the mutation does not affect the photocycle, suggesting that E142 can serve as a proton acceptor. Our simulations show that this is possible; even in the dark state, we observe up to six waters that diffuse into the periplasmic half-channel of PR within 1 μs, which are enough water molecules to bridge the 15–20 Å gap between the SB and the side chain of E142.

**Figure 4:**
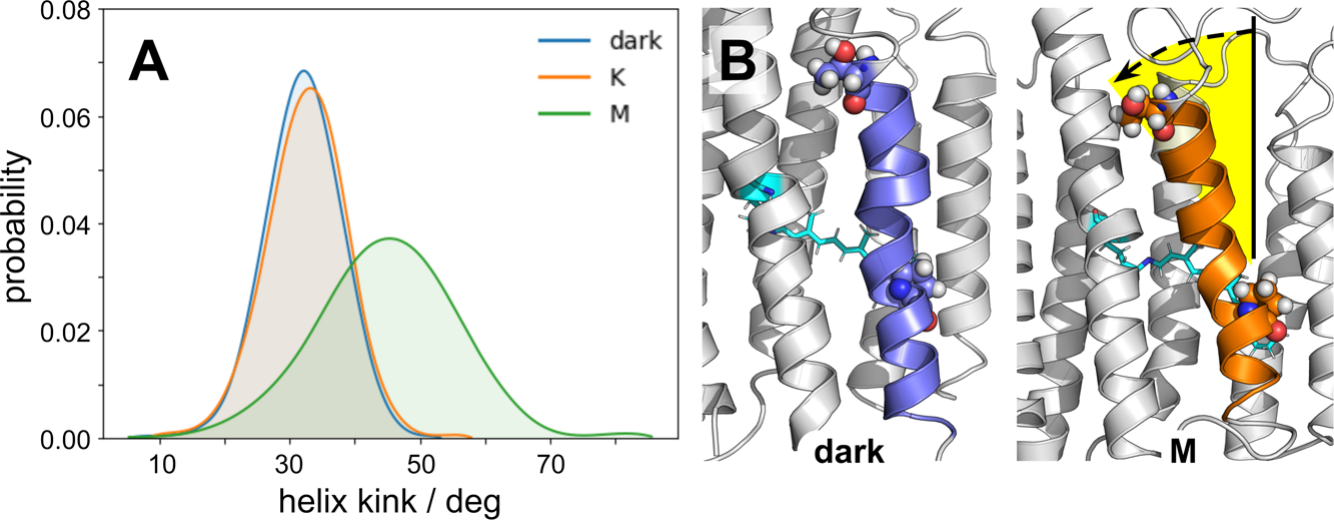
Outward movement of helix F in PR drives the M state. (A) Probability distribution of the tilt of helix F of PR with respect to the membrane normal as a function of photointermediate of the photocycle. Probability densities were rendered using a smoothing function in matplotlib. (B) Representative snapshot of helix F in an extended versus kinked conformation. *Left*: dark state; *right*: M state.

All microbial rhodopsin proton pumps undergo a characteristic outward tilt of helix F on the cytoplasmic side of the heptahelical bundle that is part of the N photointermediate.^23^ This outward tilt is a prerequisite for reprotonation of the Schiff base and regeneration of the proton donor E108. During the dark and K states, helix F remains in a more linear “closed” state, preventing an influx of bulk solvent from the cytoplasm. It is only in the M state that we observe the tilt of helix F that is associated with a marked influx of waters (Figures 4 and S4). We observed an influx of waters without this tilt in previous simulations where the proton donor E108 was deprotonated.^24,25^ This dissociation between internal hydration and tilt of helix F could be a reason why the photocycle kinetics of blue PR are an order of magnitude slower than green PR. Our observations here could also indicate that our previous simulations with a deprotonated E108 were an artifact of the system, and that the tilt here is the true behavior of blue PR.

**Figure 5:**
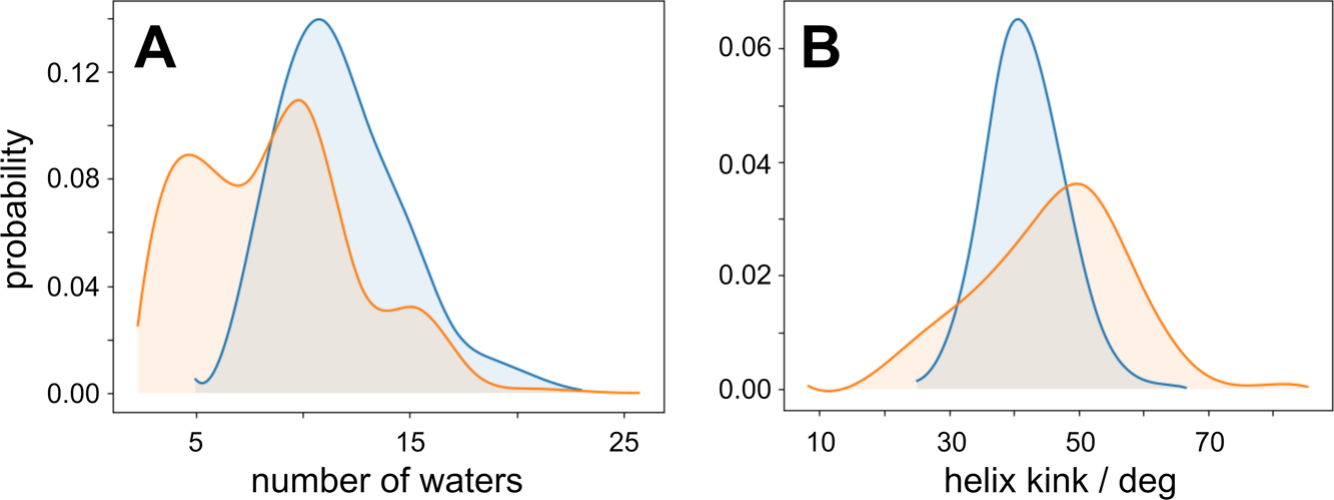
Helical formation of EF loop leads to organized hydration of the cytoplasmic half-channel and depresses tilt of helix F. (A) Probability distribution of the number of waters in the cytoplasmic half-channel of PR in the M state as a function of the EF loop adopting a coiled (*orange*) or helical (*blue*) conformation. (B) Probability distribution of helix F tilt angle in the M state as a function of the EF loop adopting a coiled (*orange*) or helical (*blue*) conformation. Probability densities were rendered using a smoothing function in matplotlib.

Adoption of secondary structure in the EF loop of GPR is a hallmark of photoactivation and onset of the M state.^61,62^ Traditionally it has been difficult to obtain resolution of the C-terminal end of helix E, presumably due to the greater flexibility of the EF loop.^21,26^ It has been hypothesized that folding into an α-helix is a necessary prerequisite to drive proton pumping, as it facilitates organization of water molecules along the proton transfer pathway in the rest of PR. This rapid interconversion, coupled with a change in hydration of the cytoplasmic half of PR, may be a mechanism by which PR can effectively prevent back-propagation of proton transfers from the Schiff base to bulk cytoplasm. When the EF loop is coiled in the M state, hydration of the cytoplasmic half-channel varies from dehydrated to nearly fully occupied (Figure 5A), indicating that the dynamic nature of the EF loop leads to suboptimal sampling of conditions that drive the M → N transition. However, upon folding into an α-helix, the tilt angle of helix F adopts a more rigid conformation, creating a pathway for proton transfer from the proton donor E108 along a water wire and to the Schiff base (Figure 5B). The stabilization of both the local protein conformation and distribution of waters within the cytoplasmic half-channel agree remarkably well with Overhauser dynamic nuclear polarization EPR studies on GPR.^62^

The EF loop also has a residue at position 178 (A178) that when mutated to arginine, induces a significant red shift in the ground state absorption.^63−65^ Previous NMR and flash photolysis studies on green PR have shown that the EF loop in PR possesses partial helicity and a beta turn, and that the A178R mutation induces loss of secondary structure that is propagated to the retinal binding pocket via an increased p*K*a of the proton acceptor D97 and changes to the interactions between the β-ionone ring and the protein.^66,67^ Although we did not directly model the A178R mutation, our results show how a change in the structural organization of the EF loop has an effect on the hydration of PR, which in turn affects the hydrogen-bonded network that is critical to facilitating proton transfer.

## Conclusions

This study represents the next step in our characterization of the photocycle of PR. Long-time scale simulations have been critical in understanding the structure–function relationship of seven-transmembrane helical proteins such as PR and G protein-coupled receptors,^68,69^ and the results in our current work show that changes to PR (both conformational and internal hydration) take on the order of microseconds to stabilize. Full hydration of the cytoplasmic and periplasmic half-channels of PR occur following the outward tilt of helix F in the M state and the rearrangement of E142. One of the key reporters on the transitions between photointermediates in the PR photocycle is the retinal chromophore; experimentally these transitions can be characterized via spectroscopic transitions, but computationally these transitions are reflected in the changes in the orientation and protonation state of retinal. Proton transfer from the Schiff base to the proton acceptor D97 is a key event in rearrangement of the water molecules within the retinal binding pocket, and a shift in the orientation of the retinal methyl groups appears to be a precursor to the larger-scale conformational changes that are characteristic of the N state. Taken together, we have provided additional support for the use of MD simulations in acquiring detailed understanding of the mechanism of microbial rhodopsins. With the recent solution of the cryo-EM structure of green PR,^21^ our future work will focus on using MD simulations to fully characterize the photocycles of both variants of PR.

## Supporting Information Available

Time-dependent plots of waters, methyl orientations, and helical tilt in all simulations (PDF)
