# Supplementary material for: Long-Time Scale Simulations Reveal Key Dynamics That Drive the Onset of the N State in the Proteorhodopsin Photocycle
Source: J Phys Chem B. 2024 Oct 10;128(42):10427–33. doi: 10.1021/acs.jpcb.4c02855 (PMC11514016; doi:10.1021/acs.jpcb.4c02855)
Supplement: Supplementary file 1 — jp4c02855_si_001.pdf [file jp4c02855_si_001.pdf]

Long-timescale simulations reveal key dynamics that drive the onset of the N state in the proteorhodopsin photocycle

Kyle R. Billings, Sadegh Faramarzi, and Blake Mertz

Corresponding author: mertz@alivexis.com

This PDF file includes:

Figs. S1 to S7

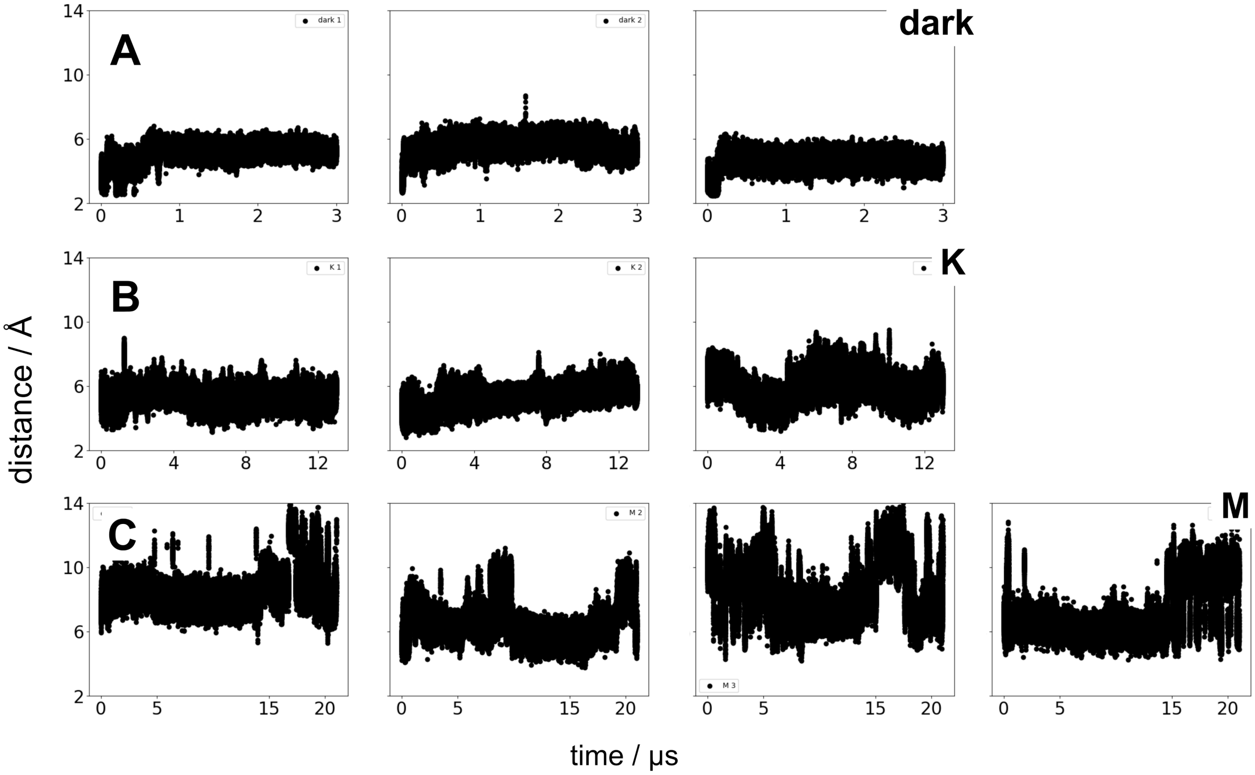

Figure S1. Time-dependent change in distances between the N of the Schiff base to the sidechain O of D97 for the dark (A), K (B), and M (C) states.

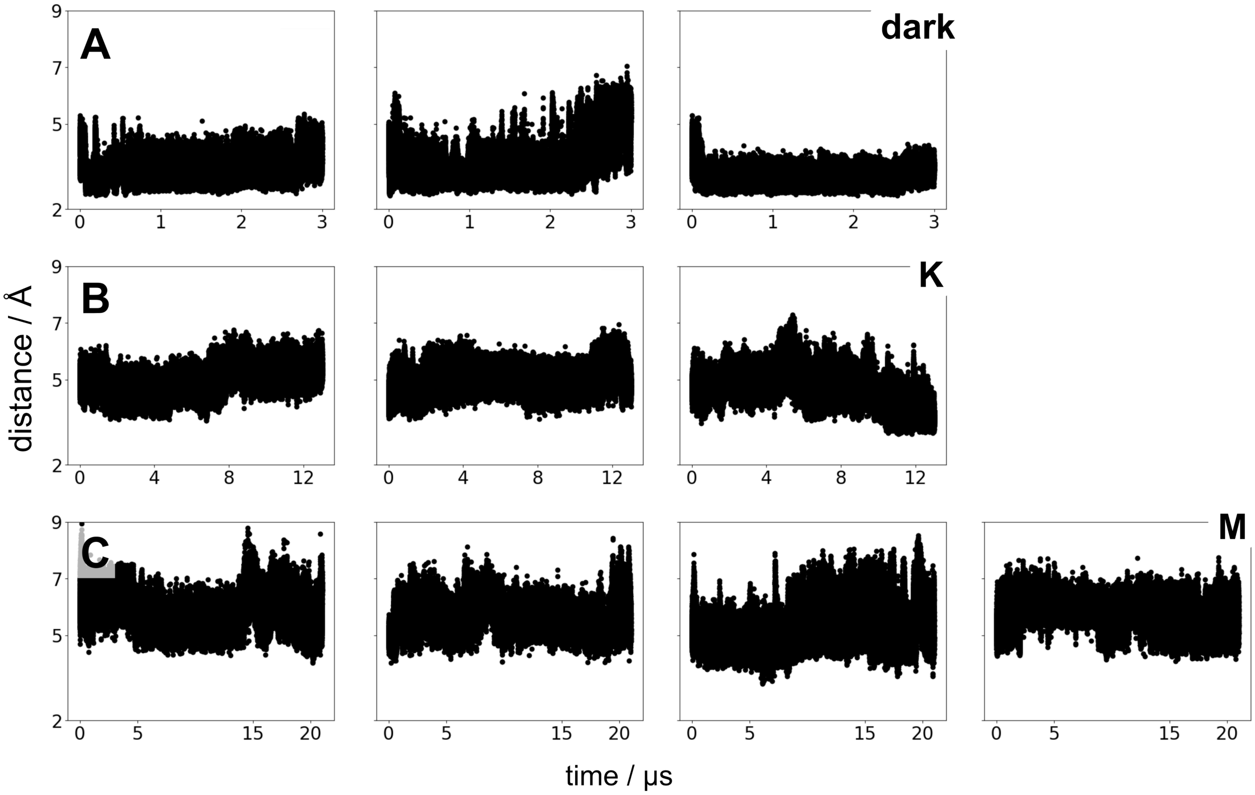

Figure S2. Time-dependent change in distances between the SB N and the sidechain O of D227 for the dark (A), K (B), and M (C) states.

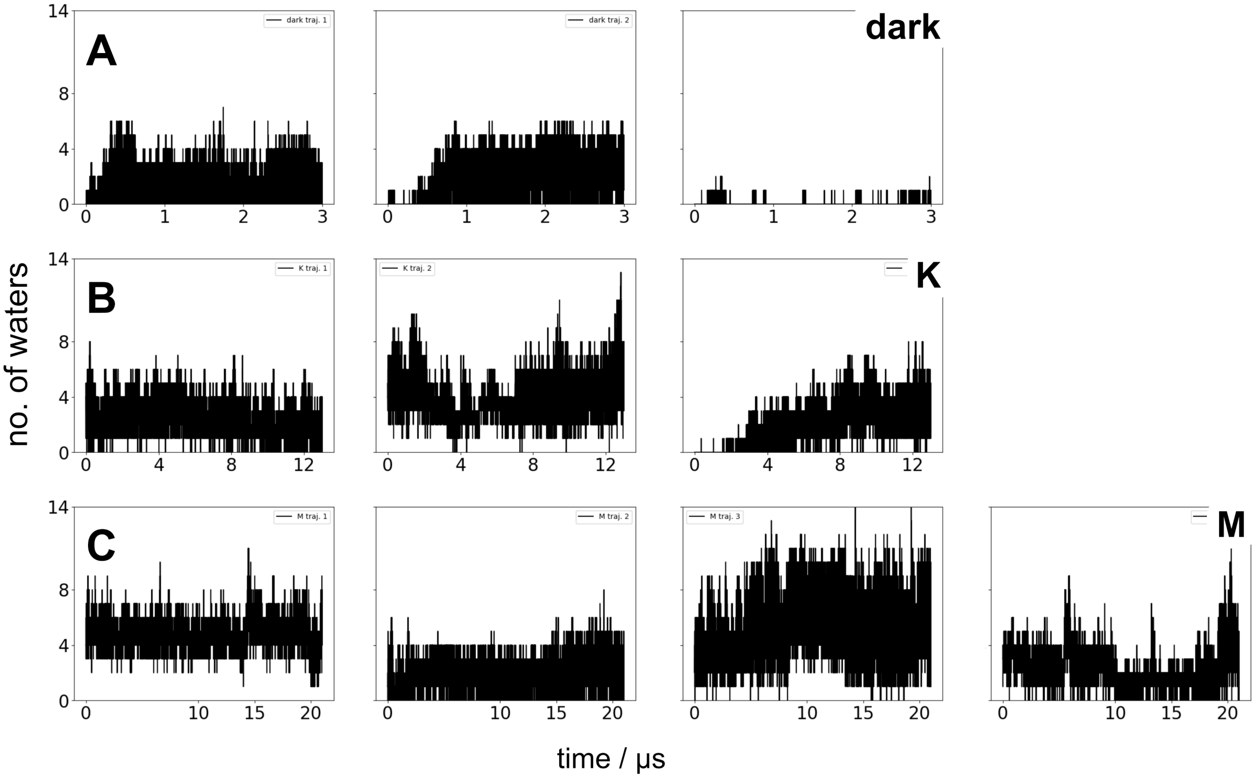

Figure S3. Time-dependent change in the number of waters within 5 Å of the carboxyl group of D97 and the NZ atom of RET for the dark (A), K (B), and M (C) states.

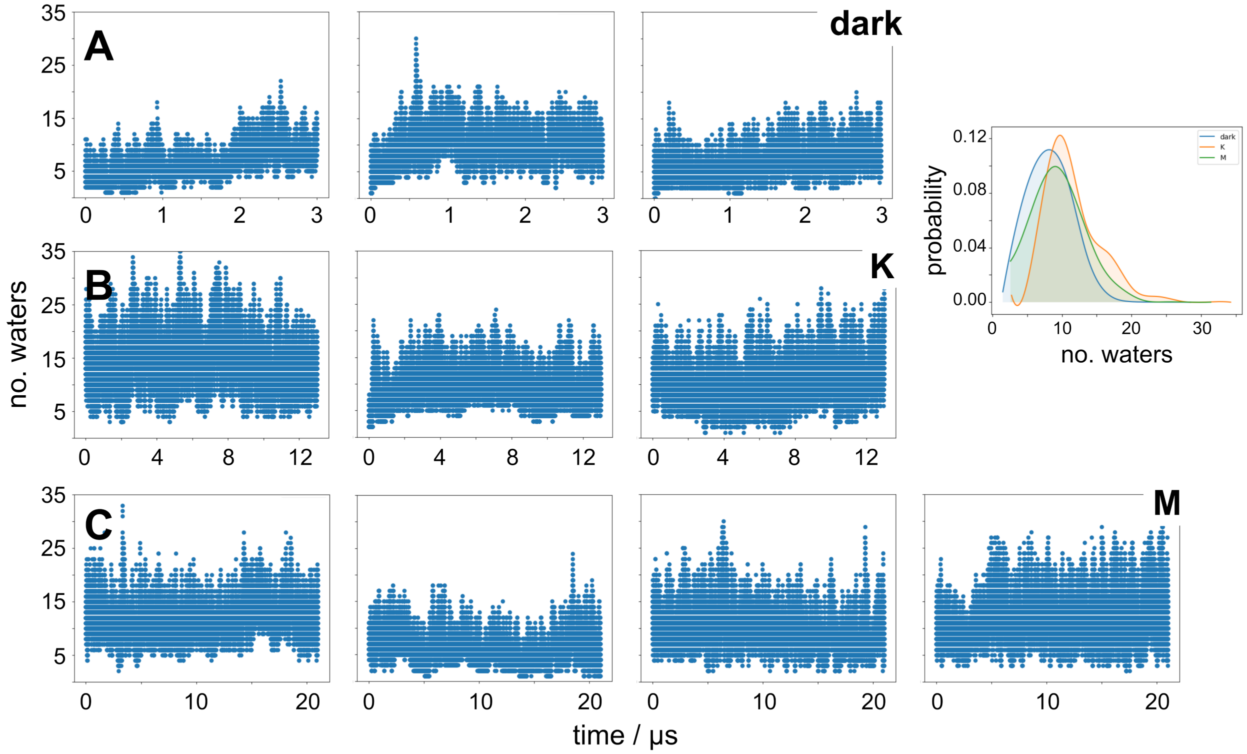

Figure S4. **Hydration of the cytoplasmic half-channel is generally stable regardless of photointermediate.** **A)** Time-dependent change of the number of waters in the cytoplasmic half-channel of PR in the dark state. **B)** Time-dependent change of the number of waters in the cytoplasmic half-channel of PR in the K state. **C)** Time-dependent change of the number of waters in the cytoplasmic half-channel of PR in the M state. Inset: normalized probability distribution of the number of waters in the cytoplasmic half-channel as a function of photointermediate.

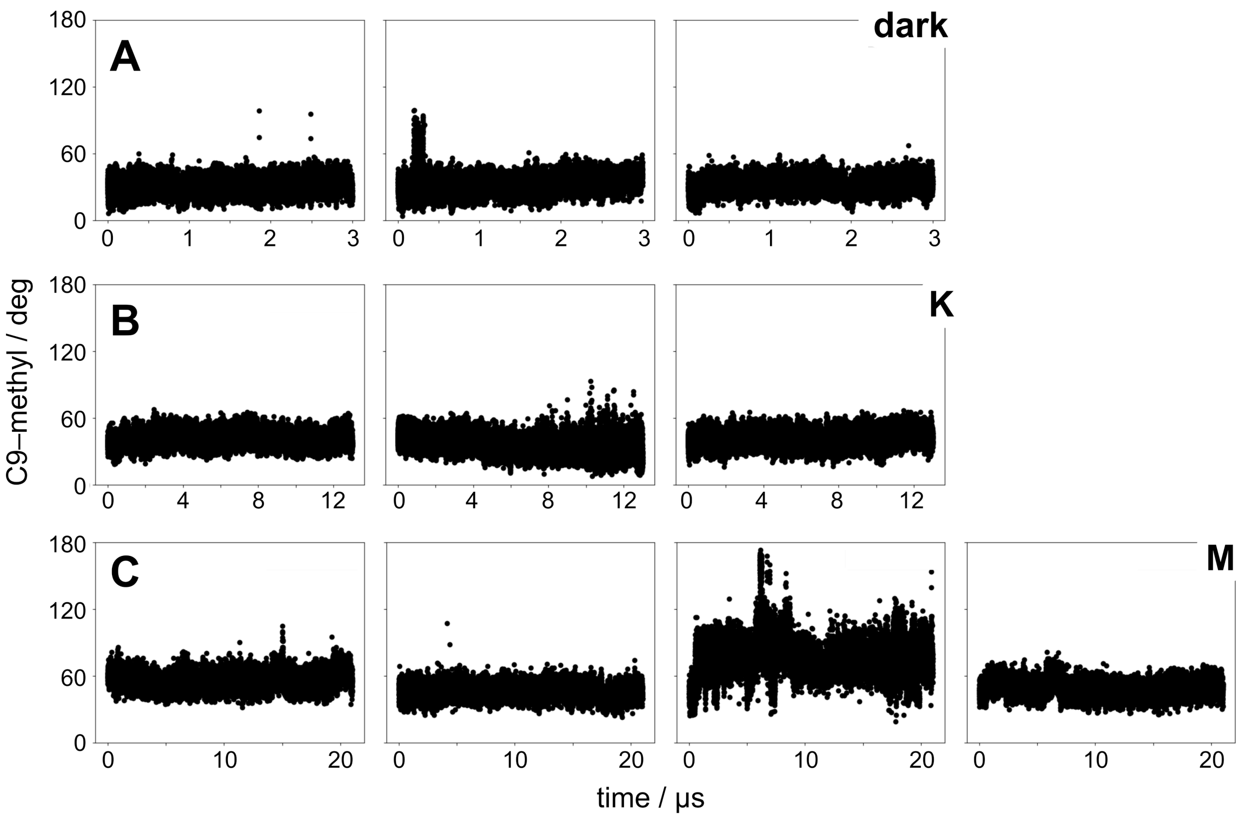

Figure S5. **Deprotonation of the Schiff base in the M state drives a slight shift in the orientation of the C9-methyl group of retinal. A)** Time-dependent change of the C9-methyl group of retinal with respect to the membrane normal in the dark state. **B)** Time-dependent change of the C9-methyl group of retinal with respect to the membrane normal in the K state. **C)** Time-dependent change of the C9-methyl group of retinal with respect to the membrane normal in the M state.

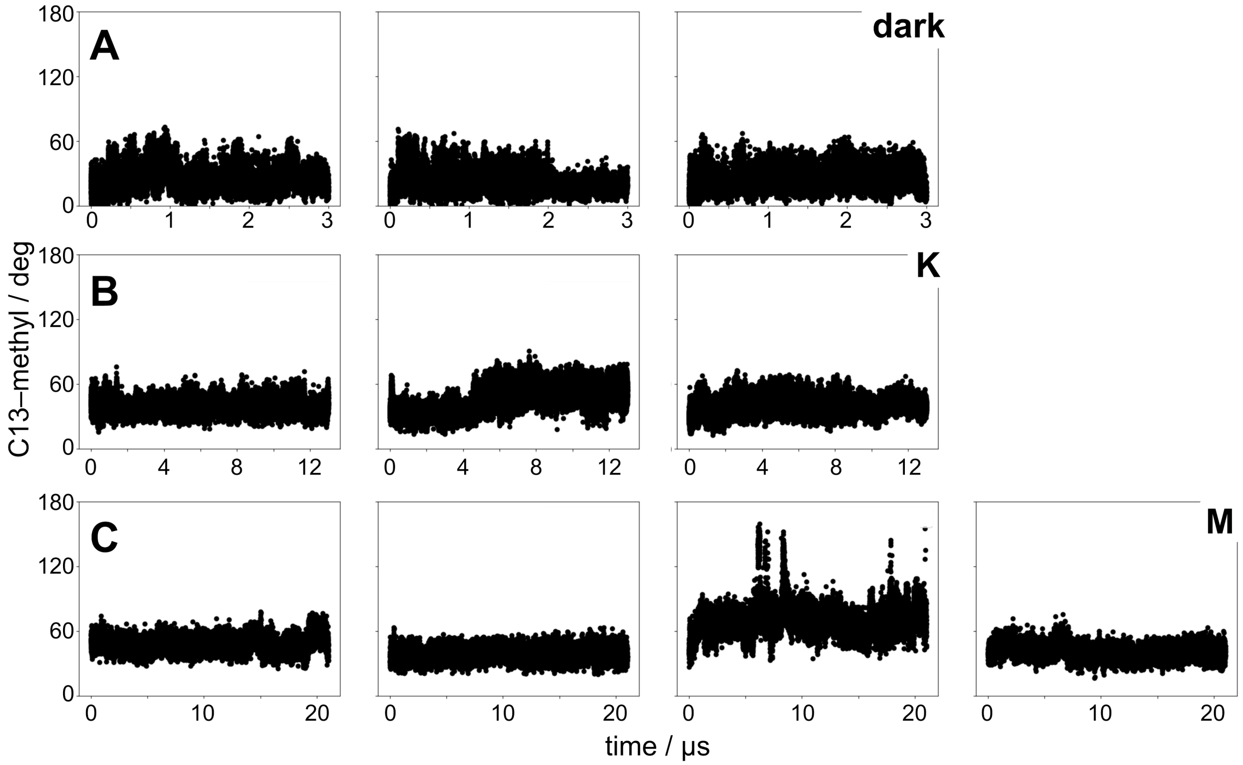

Figure S6. **The orientation of the C13-methyl of retinal is only affected by photoisomerization. A)** Time-dependent change of the C13-methyl group of retinal with respect to the membrane normal in the dark state. **B)** Time-dependent change of the C13-methyl group of retinal with respect to the membrane normal in the K state. **C)** Time-dependent change of the C13-methyl group of retinal with respect to the membrane normal in the M state.

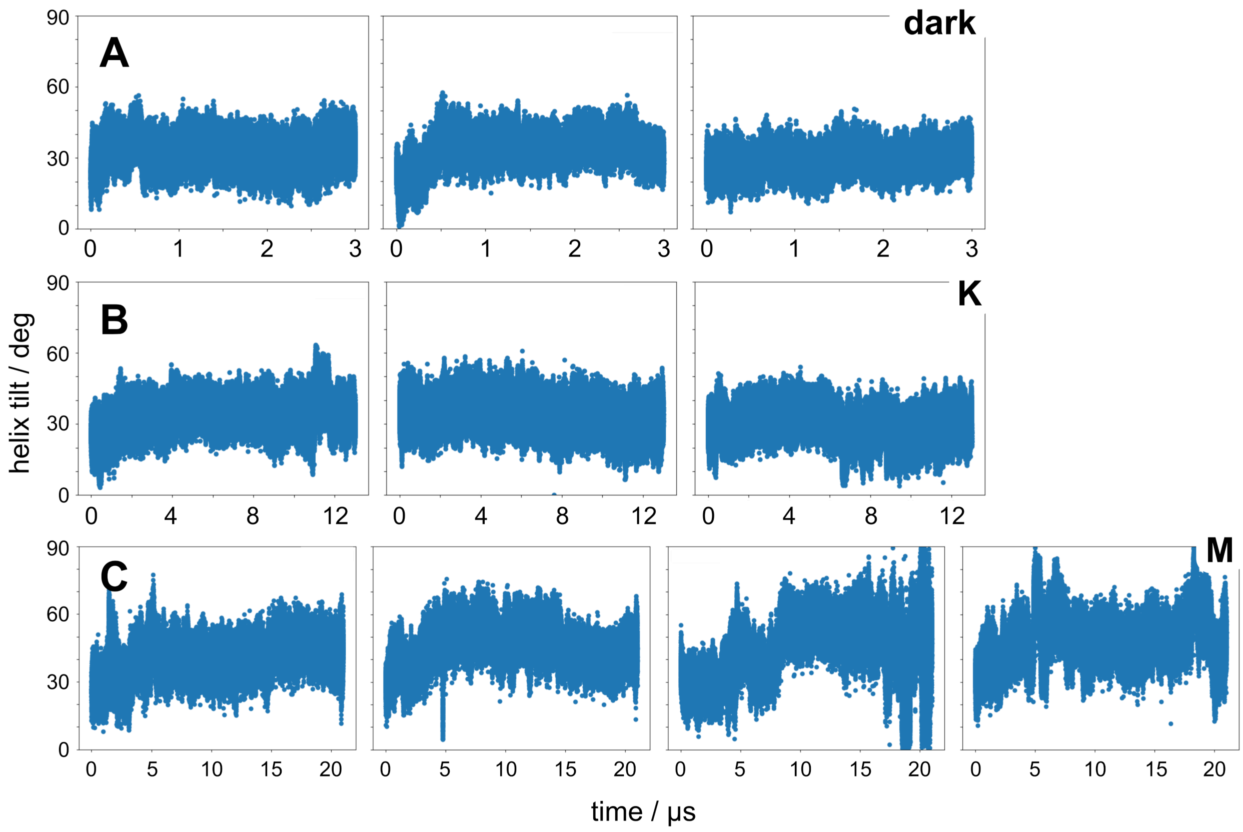

Figure S7. **Increased tilt of helix F occurs in the M state. A)** Per-trajectory time-dependent change in helix tilt of helix F of PR in the dark state. **B)** Per-trajectory time-dependent change in helix tilt of helix F of PR in the K state. **C)** Per-trajectory time-dependent change in helix tilt of helix F of PR in the M state.
